# Prevalence of interstitial lung disease among patients with systemic sclerosis in Iraqi Kurdistan

**DOI:** 10.1186/ar3614

**Published:** 2012-02-09

**Authors:** Taha Ahmad Qaradakhy, Kosar Mohamed Ali, Omer Hama Karim

**Affiliations:** 1 Department of Rheumatology, Sulaimani Internal Medicine Teaching Hospital, Sulaimani, Iraq; 2 Respiratory/General Medical Department, College of Medicine, Sulaimani, Iraq

## Background

Systemic sclerosis (SSc) associated interstitial lung disease (ILD) is the leading cause of morbidity and mortality in SSc patients.

### Aim of the study

To detect and determine the prevalence of ILD in patients with SSc in Sulaimani Governorate.

### Patients and methods

A sample of thirty patients with SSc (whom fulfilled the American Rheumatism Association preliminary criteria for the diagnosis of SSc), were collected from Sulaimani internal Medicine teaching hospital from July 2009 to July 2010.

All patients were evaluated in a cross sectional study for the evidence of ILD, almost all patients were submitted to chest radiographs (CXR), pulmonary function tests (PFT) and oxygen saturation by pulse oximetry (Spo_2_) and high-resolution computed tomography (HRCT) scan.

## Results

Patients ages ranged from 23-68 years with mean (**45.57) **years, with female predominance 27(90%) compare to 3(10%) male.

Majority of patients had limited type of systemic sclerosis 21(70%), and 15(50%) cases had restirictive ventilatory defect. Out of the thirty patients in the study 16(53.3%) patients had evidence of ILD on HRCT.

## Conclusion

1. ILD is common among patients with SSc (dcSSc type).

2. PFT & HRCT are sensitive tools for diagnosis ILD among patients with SSc.

**Table 1:** Results of pulse oximetry both during rest and exertion, chest x-ray finding, pulmonary function test

	Frequency	Percent
**O_2 _Saturation (rest)**		
Above 92	20	66.7
Below 92	10	33.3

**O_2 _Saturation (exertion)**		
Above 92	13	43.3
Below 92	17	56.7

**CXR**		
Normal	19	63.3
Basal reticular shadowing	11	36.7

**Pulmonary function test**		
Normal	15	50.0
Restrictive	15	50.0
Obstructive	0	00.0

**Table 2:** Distribution of HRCT scans abnormalities

Variables	Frequency	Percent
**CT chest**		
Normal	14	46.7
Abnormal	16	53.3

**Fibrosis**		
No	19	63.3
Yes	11	36.7

**Traction bronchiactetic changes**		
No	19	63.3
Yes	11	36.7

**Ground glass**		
No	22	73.3
Yes	8	26.7

**Honey comb**		
No	26	86.7
Yes	4	13.3

**Figure 1:**
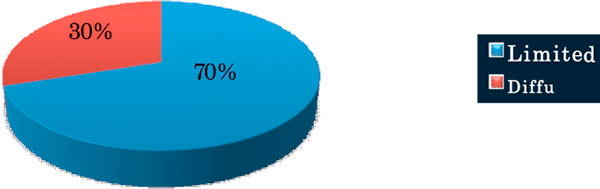
**Subsets of Systemic sclerosis**.

